# Self-management difficulties in Swedish older adults and associations with sociodemographic factors, number of conditions, depression and health status

**DOI:** 10.1080/02813432.2025.2511070

**Published:** 2025-06-01

**Authors:** Ingrid Olsson, Sabine Björk, Ulf Isaksson, Tanya Packer, George Kephart, Anna Nordström, Åsa Audulv

**Affiliations:** ^a^ Department of Nursing, Umeå University, Umeå, Sweden; ^b^ Department of Public Health and Clinical Medicine, Section of Sustainable Health, Umeå University, Umeå, Sweden; ^c^ Artic Centre, Umeå University, Umeå, Sweden; ^d^ School of Health Administration, Dalhousie University, Halifax, Canada; ^e^ School of Occupational Therapy, Dalhousie University, Halifax, Canada; ^f^ Department of Community Health and Epidemiology, Dalhousie University, Halifax, Canada; ^g^ Dept. of Medical Sciences, Rehabilitation Medicine, Uppsala University, Uppsala University Hospital, Sweden; ^h^ School of Sport Sciences, UiT The Arctic University of Norway, Tromsø, Norway

**Keywords:** Self-management, self-care, aged, chronic disease, PRISM-CC, primary health care, multimorbidity

## Abstract

**Objective:**

This study describes patterns of self-management ease and difficulty among older adults with long-term health conditions and the associations with gender, level of education, number of conditions, depression and/or health status.

**Materials and methods:**

Cross-sectional data were collected between 2021–2022 in a municipality in northern Sweden. The survey included demographic and health-related questions. To assess self-management ease or difficulty and symptoms of depression, the Patient Reported Inventory of Self-Management of Chronic Conditions (PRISM-CC) and the Geriatric Depression Scale were used. 516 older adults between 72–73 years of age with long-term health conditions were included. Descriptive statistics and logistic regression were used to describe patterns of self-management ease and difficulty and to examine which factors were associated with self-management difficulty.

**Results:**

Most older adults did not experience self-management difficulty. There were, however, differences between the seven PRISM-CC domains. The Internal domain (managing negative emotions and stress) had the highest percentage (25.39%) of older adults with self-management difficulty. In all domains, there was also a subgroup of individuals (*n* = 26) that had noticeably lower PRISM-CC scores (more difficulty). A strong association between having depressive symptoms or having poor health status and self-management difficulty was found.

**Conclusion:**

This study highlights the need for regular mental health screenings and individualized self-management support for older adults. Future research should explore intervention strategies that integrate mental health support into self-management programs for individuals with long-term health conditions.

## Introduction

Older adults (Individuals ≥ 65 years of age) [1] have a high prevalence of long-term health conditions and multi-morbidity [2,3]. Due to the considerable heterogeneity among older adults [4], individual care needs can widely differ [5,6]. Regardless of care needs, most of the daily management of long-term health conditions is carried out by older adults and their close relatives [7]. Therefore, supporting older adults to manage long-term health conditions at home is increasingly important. Self-management is recognized as a key component of living well with a long-term health condition [8–11], and adequate self-management support has shown to lead to better health outcomes and increased quality of life [12]. Self-management can be defined as ‘the intrinsically controlled ability of an active, responsible, informed and autonomous individual to live with the medical, role and emotional consequences of his chronic condition(s) in partnership with his social network and the healthcare provider(s)’ [13]. Self-management has shown to be multi-dimensional [12–14] and includes well-known aspects of managing life when living with a long-term health condition, such as medical management and monitoring of symptoms, lifestyle changes and communication with health care providers, but also incorporates, for example, access to social support and managing emotions [14,15]. This is captured by the Taxonomy of Everyday Self-management Strategies (TEDSS) framework which conceptualizes self-management as seven separate, but interrelated domains: five goal-oriented domains (Internal, Social Interaction, Activities, Healthy Behaviours and Disease Controlling) and two support-oriented domains (Process and Resource) (Table 1) [14].

**Table 1.** The taxonomy of everyday self-management strategies (TEDSS) framework; definitions and examples of strategies for the seven domains [14,15].

Domain	Definition	Example of strategies
**Resource Strategies**	Proactively seeking, pursuing and/or managing needed formal or informal supports and resources.	Self-advocating for one’s rights Navigating the healthcare system Seeking and managing social and/or community-based support
**Process Strategies**	Strategies used to be well informed and to make good decisions. Often used to support the use of other non-process strategies.	Making informed decisions Seeking information Problem-solving and finding new solutions
**Internal Strategies**	Preventing and managing stress, negative emotions and internal distress; creating inner calm.	Finding meaning and perspective in life Trying to stay positive Expressing emotions by crying or talking to a friend
**Activities Strategies**	Finding ways to participate in everyday activities (leisure activities, work activities, household chores) despite problems such as fatigue, pain, memory loss or disability.	Planning the day and make time for important activities Using aids to organise information and/or facilitate activities
**Social Interaction Strategies**	Managing social interactions and relationships to be able to participate without exposure to negative reactions.	Priorititizing and investing in important relationships Using humour to dedramatise social situations Deciding what and to whom you want to tell about your condition
**Healthy Behaviour Strategies**	Maintaining a healthy lifestyle in order to enhance health and limit the risk of lifestyle related illness.	Exercising physically and mentally to keep fit Maintaining healthy eating and sleeping habits
**Disease Controlling Strategies**	Preventing, controlling and limiting symptoms, complications and/or disease progression.	Taking medications Tracking symptoms Controlling complications by, for example, having the annual flu shot

For older adults, self-management can become increasingly challenging due to the complex and dynamic interaction between disease management, the ageing process and changing life circumstances such as bodily changes and/or loss of family and friends [16–20]. Many older adults also experience multi-morbidity, adding additional complexity. Multi-morbidity often results in fragmented care with multiple health providers following different disease-specific guidelines [11], contributing to a high treatment burden [21] and even conflicting treatment recommendations [16]. Having multi-morbidity is strongly associated with increased health care utilization [22,23] and decreased health-related quality of life [23,24]. High disease and symptom burden has been shown to negatively affect self-management [25,26]. Limited education [27], low health literacy [28,29], gender [26,27], and depression [30] have also been associated with self-management difficulties. Few studies have, however, looked specifically at older adult populations and some studies, on gender for example, have shown mixed results [26,27].

Primary health care is a key setting to support older adults in self-management [31], for which person-centered and holistic care, together with continuity of care, are pivotal [17,32]. Due to individual disease trajectories, life contexts, existing support, and individual abilities [15,33,34], the need for self-management support is highly individual and can change over time. Still, the greatest emphasis, both in primary health care and in self-management interventions, has been on medical management and promoting healthy behaviors [26,35], often neglecting the role and emotional consequences of living with a long-term health condition [35]. Moreover, rising health utilization and associated costs [36], combined with staff shortages [37,38], make it essential to use existing resources efficiently, and primary health care professionals often have to prioritize between different patient needs [39]. The ability to identify which older adults need self-management support and pinpoint the specific nature of their difficulties provides an opportunity to deliver feasible, person-centered, and tailored self-management support more efficiently. In order to do that, information regarding the extent to which older adults with long-term health conditions experience difficulties with self-management, the specific nature of those difficulties, and their associated factors [19,40] need to be described. Therefore, this study aimed to describe patterns of self-management ease and difficulty among older adults with long-term health conditions by answering the following research questions:

1. Do older adults with long-term health conditions find self-management difficult, and if so, do these difficulties vary across the different self-management domains outlined in the TEDSS framework?
2. Is there an association between self-management difficulty and gender, level of education, number of conditions, depression and/or health status? Furthermore, do these associations vary across the TEDSS domains?

## Materials and methods

### Study design, setting and participants

Participants were recruited through the Healthy Aging Initiative (HAI) study [41], a long-term research project that tracked health outcomes in older adults in Umeå municipality between the years 2012–2023. All residents in the municipality were invited to participate in the HAI study the year they turned 70 years of age. The approximate participation rate in the HAI study was high (84%). For this study, we contacted all HAI study participants enrolled during 2018 and 2019 (*n* = 1117), making them 72-73 years of age at the time of the data collection. Cross-sectional survey data for the current study were collected between May 2021 and February 2022 (See Appendix 1, flowchart of recruitment). Potential participants received an invitation to complete a survey either online or by paper-and-pencil. The invitation letter included, for example, a description of the project and its purpose, inclusion criteria for the study, a short explanation of the different parts included in the survey, information regarding data management, and research consent practices. Since participants’ health status was not fully known, the survey was sent to all HAI participants. However, the information letter specified that having one or more long-term health conditions was an inclusion criterion, so older adults without long-term health conditions did not complete the survey. Two reminders were sent two weeks and four weeks after the initial invitation. Of the 1117 invited HAI study participants, 48.5% (*n* = 542) returned the survey and had one or more long-term health conditions. This proportion is consistent with previous studies on the prevalence of long-term health conditions in this age group [3,42].

Ethical approval for the HAI study was received from the Swedish Ethical Review Authority and the Regional Ethics Review Board in Umeå in 2007 (Dnr 2012-85-32 M- and 07-031 M), and a complementary ethical application for this project was approved in 2020 (Dnr 2020-02387). All participants gave written informed consent.

### Survey

The survey included questions on the number and type of self-reported long-term health conditions, demographic characteristics (such as gender and highest level of education), and standardized measures to assess self-management ease and difficulty and symptoms of depression. To assess health status, participants answered the question: ‘How do you rate your general health?’ with a four-option response scale: ‘very poor’, ‘poor’, ‘good’ and ‘very good’.

### The patient-reported inventory of self-management of chronic conditions (PRISM-CC)

Self-management ease or difficulty was assessed using the Swedish version of the Patient Reported Inventory of Self-Management of Chronic Conditions (PRISM-CC). The PRISM-CC is a new, generic, multi-dimensional instrument that measures self-perceived self-management ease or difficulty within the seven TEDSS domains [43]. The Swedish version of PRISM-CC has shown good structural validity, test-retest reliability [44], and measurement equivalence [45] and the data in this study has previously been used to validate the PRISM-CC. The PRISM-CC consists of 36 items (4–8 per domain) formulated as statements (Appendix 2), with a six-option response scale [1–6]. Scores are calculated per domain, with low scores indicating more self-perceived self-management difficulty.

### Geriatric depression scale, 15-item (GDS-15)

To assess symptoms of depression, the Swedish version of the Geriatric Depression Scale (GDS-15) was used. GDS-15 was chosen for its brevity and suitability in detecting depression among older adults [46] and has shown to be a useful screening tool [47]. GDS-15 consists of 15 items with ‘Yes’ or ‘No’ answers. Total scores range from 0 to 15, with a recommended cut-off of ≥6 indicating depression [48].

### Statistical methods

**Figure 1.**
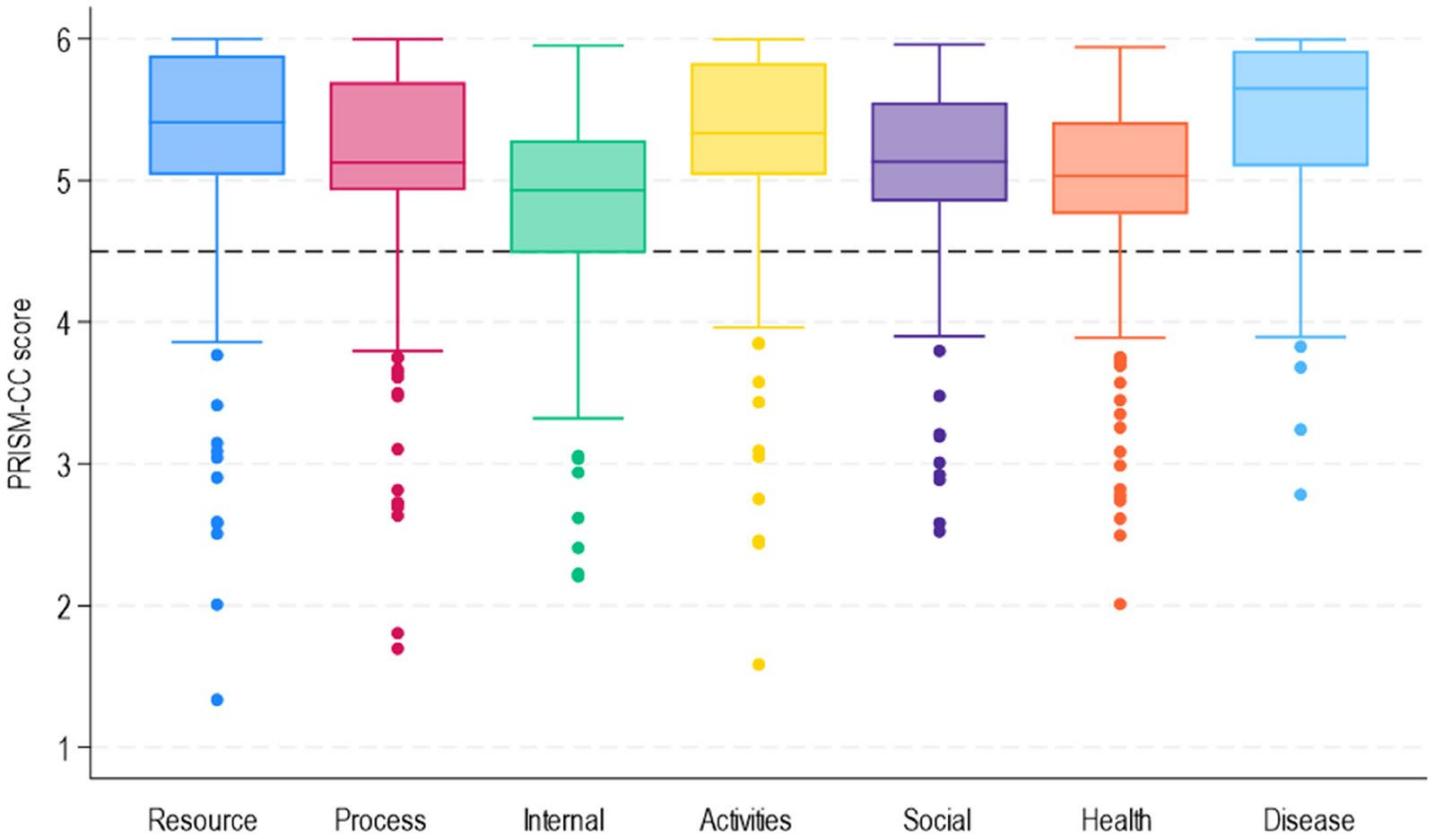
Boxplots of PRISM-CC scores per domain. The dotted line shows the cut-off for self-management difficulty in the respective domain. The outliers were identified as 26 separate individuals.

Stata version 18 [49] was used for all analyses. Individuals with more than 50% missing or not applicable (NA) answers in the PRISM-CC section of the survey were excluded (*n* = 26), leaving 516 individuals for inclusion. First, the median and quartile scores for each PRISM-CC domain were calculated. PRISM-CC domain scores were then dichotomized into having ‘no difficulty’ (domain scores >4.5) and ‘difficulty’ (domain scores ≤4.5). This cut-off was chosen based on the response options, where response options 1–4 included the word ‘difficulty’ and options 5–6 the word ‘easy’. Once dichotomized, the number of individuals with self-management difficulty per domain was calculated. The boxplot illustrating the distribution of PRISM-CC scores per domain revealed outliers (Figure 1). Those were identified as being a subpopulation of 26 separate individuals with the lowest PRISM-CC scores (more difficulty with self-management). The subpopulation was included in the analyses of the total sample and examined separately using descriptive statistics.

Logistic regression with purposeful selection [50] was used to examine the association between self-management difficulty and multiple factors, including gender, level of education, comorbidities, depression, and health status. The analysis was conducted by domain. The dependent variable was self-management difficulty (yes or no). For the independent variables, number of conditions was categorized into having one, two, three or four or more diseases. Education was dichotomized into ‘Lower education’ (i.e. ≤high school) and ‘Higher education’ (i.e. ≥bachelor’s degree). Health status was dichotomized into ‘poor health’ if participants stated their health as poor or very poor and ‘good health’ if participants stated their health as good or very good. GDS-15 scores were dichotomized according to the recommended cut-off of ≥6 into ‘no signs of depression’ or ‘signs of depression’.

First, simple logistic regressions with each independent variable (i.e. gender, level of education, number of conditions, GDS-15 scores, and health status) were conducted. All independent variables with a p-value <0.25 were kept as potential correlated variables. This cut-off in p-value was chosen to avoid removing variables that could indirectly affect the outcome variable and would be missed if using the more common p-value of <0.05 [50]. A first multivariable model was then fitted, including all the potential variables. The importance of each variable was assessed using Wald statistics. Variables that did not contribute at a significance level <0.05 and/or did not significantly change the coefficients of the other variables (>20%) were removed. The new, smaller model was compared to the old model using the partial likelihood ratio test. All excluded variables from the first step were then added back to the model, one by one, to ensure they did not contribute to the model [51]. Finally, interactions and multicollinearity between the included variables and model adequacy were assessed for the final model. Bootstrapping with 100 replications was used to verify the results [52].

## Results

For participant characteristics, see Table 2. Participants demonstrated various self-reported long-term health conditions, the most common being cardiovascular disease (84.48%), muscular-skeletal disease (58.62%), and respiratory disease (31.03%). The frequency of self-reported depression was 3.68%. This is lower than the frequency of individuals with a GDS-15 score ≥6, which was 21.32%.

**Table 2.** Demographic and clinical characteristics of total sample (*n* = 516) and subpopulation experiencing most self-management difficulty (*n* = 26).

	Total sample (*n* = 516)	Subpopulation experiencing most self-management difficulty (*n* = 26)
Characteristic	n (%)	n (%)
**Gender**		
Female	256 (49.61)	14 (53.85)
Male	249 (48.26)	12 (46.15)
Missing	11 (2.13)	0
**Living situation**		
Live alone	114 (22.09)	5 (19.23)
Shared household	390 (75.58)	21 (80.77)
Missing	12 (2.33)	0
**Marital status**		
Married/cohabitant	386 (74.81)	20 (76.92)
Living apart together	12 (2.33)	0
Widow/widower	29 (5.62)	2 (7.69)
Single	74 (14.34	4 (15.38)
Missing	15 (2.91)	0
**Highest level of education completed **		
Elementary school or less	105 (20.35)	13 (50.00)
High school	158 (30.62)	13 (50.00)
Graduate degree	235 (45.54)	0
Missing	18 (3.49)	0
**Economic difficulties in the past year**		
Yes	14 (2.71)	3 (11.54)
No	494 (95.74)	23 (88.46)
Missing/Do not wish to answer	8 (1.55)	0
Number of long-term health condition(s)*		
1	116 (22.48)	3 (11.54)
2	144 (27.91)	3 (11.54)
3	127 (24.61)	7 (26.92)
4 +	129 (25.00)	13 (50.00)
**GDS-15 scores**		
<6	406 (78.68)	10 (38.46)
≥6	110 (21.32)	16 (61.54)
**Health status**		
Good	451 (87.40)	12 (46.15)
Poor	51 (9.88)	14 (53.85)
Missing	14 (2.71)	0
* = participants may have more than one condition.		

### Patterns of self-management difficulty across the different TEDSS domains

**Figure 2.**
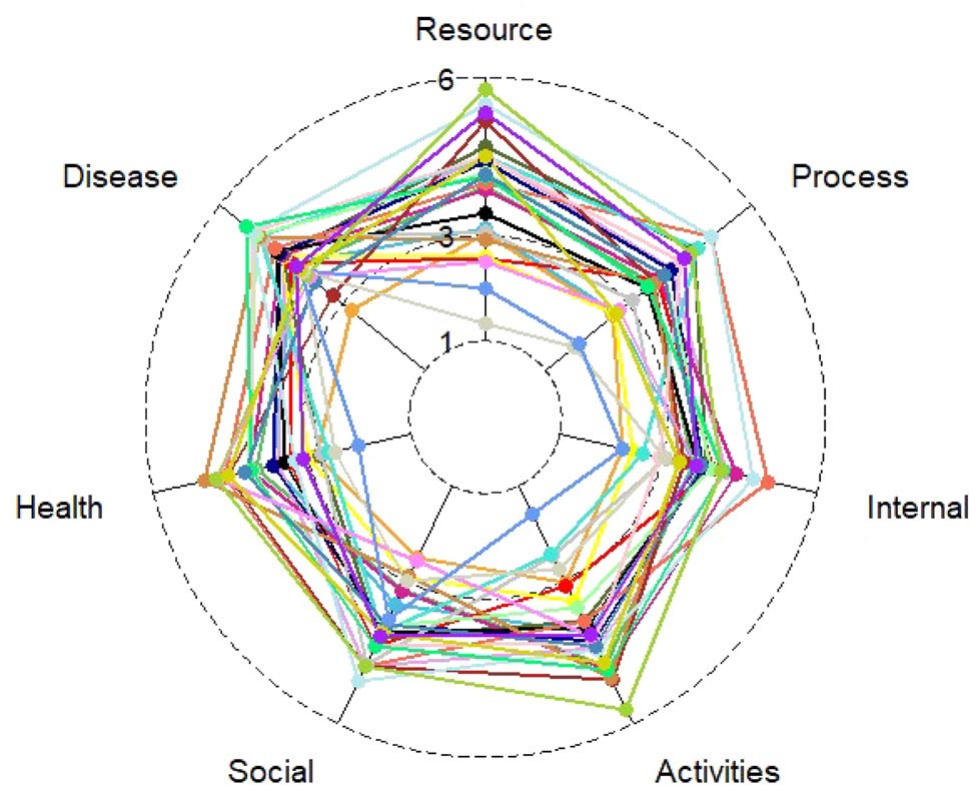
The PRISM-CC scores of the 26 individuals with most self-management difficulty in one or more domains. Each colour represents one individual. The scores range from 1-6, with lower scores indicating more self-management difficulty. The figure shows that those individuals tended to experience difficulty in more than one domain.

Overall, the PRISM-CC scores were high in all domains (Table 3). The percentage of older adults experiencing self-management difficulty ranged from 7 to 25%, depending on the domain. The highest percentage (25.39%) experienced difficulty managing negative emotions and stress (the Internal domain). The lowest proportion of people experienced difficulty seeking and managing formal and informal resources (the Resource domain). In all domains, some individuals scored considerably lower than the average for that domain and showed as outliers (*n* = 26) (Figure 1). All individuals shown as outliers had difficulty in more than one domain, and most experienced difficulty in several domains (Figure 2). Those individuals seemed to have overall lower education, more long-term health conditions, be more likely to have symptoms of depression, and have poorer self-reported health than the total sample, even though this could not be verified statistically due to the small sample (Table 2). This is also seen in the full sample, and illustrated by Table 4, where the pattern of self-management difficulty across TEDSS domains is displayed. Interestingly, 75-96% of all individuals with self-management difficulty in one or more domains also had difficulty in managing negative emotions and stress (the Internal domain).

**Table 3.** Median and quartiles of PRISM-CC scores, together with the number of individuals experiencing self-management difficulty per domain.

Domain	Median (Q1–Q3)*	Number with self-management difficulty per domain(%)
Resource	5.41 (5.04–5.88)	34 (6.59)
Process	5.13 (4.93–5.69)	58 (11.24)
Internal	4.93 (4.49–5.28)	131 (25.39)
Activities	5.33 (5.04–5.83)	42 (8.14)
Social interaction	5.13 (4.85–5.55)	50 (9.69)
Healthy Behavior	5.03 (4.76–5.41)	78 (15.12)
Disease Controlling	5.65 (5.10–5.92)	50 (9.69)

*Median and quartiles are calculated of the total sample. Q1= Lower quartile, Q3= Upper quartile.

**Table 4.** Patterns of self-management difficulty across TEDSS domains.

	Number of individuals with self-management difficulty per domain						
Difficulty other TEDSS domains*	Resource (*n* = 34)	Process (*n* = 58)	Internal (*n* = 131)	Activites (*n* = 42)	Social (*n* = 50)	Health (*n* = 78)	Disease (*n* = 50)
Resource	…	27	31	17	24	19	15
	…	46.55%	23.66%	40.48%	48.00%	24.36%	30.00%
Process	27	…	51	24	32	31	27
	79. 41%	…	38.93%	57.14%	64.00%	39.74%	54.00%
Internal	31	51	…	34	48	59	41
	91.18%	87.93%	…	80.95%	96.00%	75.64%	82.00%
Activities	17	24	34	…	21	36	14
	50.00%	41.38%	25.95%	…	42.00%	46.15%	28.00%
Social	24	32	48	21	…	28	23
	70.59%	55.17%	(36.64%)	50.00%	…	35.90%	46.00%
Health	19	31	59	36	28	…	18
	55.88%	53.45%	45.04%	85.71%	56.00%	…	36.00%
Disease	15	27	41	14	23	18	…
	44.12%	46.55%	31.30%	33.33%	46.00%	23.08%	…

*Calculated from the number of individuals with self-management difficulty per domain.

### Associations between self-management difficulty and gender, level of education, number of conditions, depression and health status

Results of the univariate analyses (Table 5), showed that, in all TEDSS domains, having symptoms of depression or having poor health status were significantly associated with self-management difficulty. Both depression and having poor health status were most strongly associated with participation in everyday activities (Activity domain). Individuals with symptoms of depression had 5.38 (2.81–10.32) times the odds, and individuals with poor health status 27.95 (13.16-59.32) times the odds of having self-management difficulty compared to those without symptoms of depression or with good health status. Furthermore, compared to having only one disease, having four or more conditions was associated with self-management difficulty in four domains (Process, Internal, Activity and Healthy Behaviors). Gender and level of education showed no significant association with self-management difficulty in the univariate analysis.

**Table 5.** Univariate and multivariate logistic regression per domain for outcome variable self-management difficulty (yes/no).

	Univariate analysis						
	Resource	Process	Internal	Activity	Social Interactions	Healthy Behaviours	Disease Controlling
**Gender**							
P-value	0.415	0.756	0.121*	0.582	0.430	0.704	0.313
Odds ratio (95% CI)	0.74 (0.36-1.52)	0.92 (0.53-1.59)	0.73 (0.05-1.09)	0.84 (0.44-1.58)	0.79 (0.44-1.42)	1.10 (0.67-1.79)	1.36 (0.75-2.48)
**Number of conditions (1, 2, 3, 4+)**							
**2)** P-value	0.701	0.934	0.184*	0.190*	0.457	0.507	0.457
Odds ratio (95% CI) 2vs1	0.80 (0.25-2.54)	0.96 (0.40-2.32)	0.66 (0.36-1.22)	0.39 (0.095-1.59)	0.71 (0.29-1.74)	1.33 (0.58-3.04)	0.71 (0.29-1.74)
**3)** P-value	0.537	0.991	0.530	0.707	0.656	0.241*	0.693
Odds ratio (95% CI) 3vs1	1.40 (0.48-4.06)	1.01 (0.41-2.46)	1.21 (0.67-2.16)	1.23 (0.41-3.66)	0.82 (0.33-2.00)	1.64 (0.72-3.74)	1.18 (0.51-2.72)
**4)** P-value	0.159*	0.019**	0.035**	0.002**	0.214*	<0.001**	0.587
Odds ratio (95% CI) 4vs1	2.05 (0.75-5.60)	2.55 (1.17-5.57)	1.83 (1.04-3.2)	4.41 (1.74-11.17)	1.65 (0.75-3.63)	3.95 (1.85-8.40)	1.26 (0.55-2.86)
**Level of education**							
P-value	0.607	0.273	0.826	0.831	0.859	0.177*	0.421
Odds ratio (95% CI)	0.83 (0.41-1.68)	1.37 (0.78-2.40)	0.96 (0.64-1.43)	0.93 (0.49-1.77)	1.05 (0.59-1.90)	1.41 (0.10-2.33)	1.28 (0.71-2.34)
**Depression**							
P-value	<0.001**	<0.001**	<0.001**	<0.001**	<0.001**	<0.001**	0.003**
Odds ratio (95% CI)	4.77 (2.34-9.71)	3.32 (1.88-5.88)	4.83 (3.07-7.57)	5.38 (2.81-10.32)	4.08 (2.23-7.45)	3.44 (2.06-5.74)	2.53 (1.37-4.67)
**Health status**							
P-value	<0.001**	<0.001**	<0.001**	<0.001**	<0.001**	<0.001**	<0.001**
Odds ratio (95% CI)	8.15 (3.81-17.45)	11.26 (5.85-21.66)	7.80 (4.17-14.59)	27.95 (13.16-59.32)	5.28 (2.62-10.62)	10.72 (5.71-20.13)	4.50 (2.22-9.10)
	**Multivariate analysis**						
**Number of conditions (1, 2, 3, 4+)**							
**2)** P-value				0.170		0.410	
Odds ratio (95% CI) 2vs1				0.34 (0.08-1.58)		1.45 (0.60-3.49)	
**3)** P-value				0.491		0.730	
Odds ratio (95% CI) 3vs1				0.64 (0.18-2.27)		1.17 (0.47-2.93)	
**4)** P-value				0.211		0.048**	
Odds ratio (95% CI) 4vs1				1.99 (0.68-5.88)		2.31 (1.01-5.30)	
**Level of education**							
P-value						0.190	
Odds ratio (95% CI)						1.46 (0.83-2.57)	
**Depression**							
P-value	0.003**	0.060	<0.001**	0.048**	0.003**	0.013**	0.047**
Odds ratio (95% CI)	3.19 (1.47-6.91)	1.88 (0.98-3.64)	3.66 (2.49-5.96)	2.29 (1.01-5.22)	2.77 (1.43-5.35)	2.17 (1.18-4.00)	1.97 (1.01-3.86)
**Health status**							
P-value	<0.001**	<0.001**	<0.001**	<0.001**	<0.001**	<0.001**	0.001**
Odds ratio (95% CI)	5.40 (2.39-12.22)	9.07 (4.55-18.08)	5.47 (2.83-10.58)	18.90 (8.21-43.50)	3.65 (1.73-7.72)	7.34 (3.71-14.53)	3.49 (1.65-7.41)
R²	0.135	0.155	0.133	0.333	0.090	0.157	0.058
Chi2	<0.001	<0.001	<0.001	<0.001	<0.001	<0.001	<0.001
Walds test:							
Depression/ health status	<0.001	<0.001	<0.001	<0.001	<0.001	<0.001	<0.001
Number of conditions						0.1988	

Female gender, higher education and having good health are reference groups. CI = Confidence Interval. *P-value <0.25, ** Significant p-value <0.05.

In the multivariate analysis, depressive symptoms were significantly associated with self-management difficulty in six of the seven TEDSS domains. The strongest association was with difficulty in seeking and managing formal and informal resources (Resource domain) and managing negative emotions and stress (Internal domain), where individuals with depression had about three times the odds of having self-management difficulty compared to individuals without symptoms of depression. In all domains, having poor health status was associated with having more difficulty with self-management. The strongest association was with participation in everyday activities (Activity domain), where individuals with poor health status had 18.90 (8.21-43.50) times the odds of having self-management difficulty compared to those with good health status. In the Healthy Behaviors domain (maintaining a healthy lifestyle), having four or more conditions was associated with more self-management difficulty compared to individuals with only one condition (odds ratio 2.31 (1.01-5.30)).

## Discussion

This study provides insight into self-management patterns among older adults with long-term health conditions, uncovering important associations with depression and health status. Encouragingly, most older adults in this sample did not experience self-management difficulties. There can be several reasons for this. First, not all long-term health conditions alter peoples’ everyday lives and demand complex self-management regimens. For instance, conditions with minimal symptom burden are related to less engagement in self-management [53]. Second, older adults who have lived with their condition(s) for a long time might have developed and integrated self-management regimens into their everyday lives [54]. Therefore, they could perceive even complex self-management as easily performed. Since most older adults with long-term health conditions can perform their self-management with ease, primary care initiatives should be directed to the minority of older adults that need self-management support the most, many of whom were found to have difficulty in more than one TEDSS domain. This highlights a need, within primary health care, to measure self-management difficulty to identify (1) which older adults need self-management support interventions and (2) towards what self-management domains those interventions should be directed. It is more efficient for health care providers to focus self-management support to those domains were it is most needed, as perceived by the older adult. Unfortunately, most self-management measures do not differentiate needs, leaving health providers offering standardized and one-size fits all interventions.

Over one-fourth of respondents, even in a relatively healthy sample, experienced self-management difficulty in the Internal domain, wich includes dealing with emotions and stress. Moreover, most individuals with self-management difficulty in any TEDSS domain simultaneously had difficulty in the Internal domain. This might suggest that the internal domain is particularly important for older adults’ overall self-management ability. It is well-known that living with a long-term health condition can negatively affect health-related quality of life and be emotionally demanding [17,24, 55,56]. Despite this, research shows that psychological and emotional consequences of living with a long-term health condition are rarely addressed in self-management interventions [35]. Our results suggest that more interventions to support internal self-management need to be developed. Furthermore, our findings demonstrate a strong association between depression and difficulty in self-management among older adults with long-term health conditions. Interestingly, depression was not just associated with internal self-management but with all domains except the process domain (problem-solving). Associations between depression and self-management is consistent with previous research [25,26,30,57–59]. The association might be a result of low level of agency and motivation common to people with depression and highlights the importance of routine screening for depression among older adults with long-term health conditions. It also suggests that anyone identified as being depressed should be screened for self-management difficulty in order for them to receive the support they need.

When comparing the frequency of self-reported depression with the proportion of individuals having a GDS-15 score ≥6, the results show a clear discrepancy. This might suggest that several older adults in this sample live with depression without being diagnosed. Early identification of individuals at risk for depression could enhance their self-management ability, ultimately improving their overall health outcomes. Targeted interventions, including mental health support for individuals experiencing self-management difficulty, could improve both health outcomes and quality of life for older adults with long-term health conditions. By integrating psychological support with practical self-management strategies, healthcare providers could offer more comprehensive care, addressing both the physical and emotional challenges faced by patients with long-term health conditions [60].

Further, this study showed a strong association between self-rated health status and self-management difficulty. Older adults who rated their health as poor were more likely to rate their self-management as difficult. The Odds Ratios were high; however, the confidence intervals were also wide, indicating variation in the sample. The Activities, Healthy Behavior and Process domains had particularly high Odds Ratios (e.g. 18.90 (8.21–43.50), 7.34 (3.71–14.53), 9.07 (4.55–18.08)), which could mean that older adults with poor health struggle with being able to perform activities important to them, engage in healthy behaviors and problem-solve in regards to their self-management. Due to this study’s cross-sectional design, it is not possible to ascertain the direction of the association – does poor health create specific difficulties in performing self-management or does difficulty with self-management lead to poor health? According to previous research, both are possible. Research shows that poor health can impact the ability to perform self-management [19]; persistent pain and low function can, for example, impede physical activity and taking part in activities. However, being unable to conduct suitable self-management can also impact health, since self-management has been shown to mitigate symptoms and disease complications [61]. Which ever is true, it is reasonable to suggest that older adults who perceive their health as poor need more and individually tailored, self-management support.

There are limitations to this study. First, there is a possibility that some individuals experiencing considerable self-management difficulty chose not to participate due to the perceived response burden. In addition, the long-term health conditions were self-reported, which could have affected the accuracy of the data [62]. However, the prevalence and types of long-term health conditions in our sample were similar to those found for this age group in other studies [3,42]. Second, due to limited power, there is a possibility that this study may have detected only the strongest associations, therefore, conclusions about which variables are not associated with self-management difficulty should be interpreted with caution. For example, we found no association between education and self-management difficulty. However, in the subsample of participants (*n* = 26) having severe self-management difficulty, a larger proportion appeared to had lower education levels than the total sample. A sample with a larger number of participants with lower levels of education might have confirmed the previously reported association between education, health literacy and self-management difficulty [27–29]. Furthermore, the proportion of individuals with post-secondary education was higher in this study than the Swedish average. This is, however, common in municipalities with a strong academic culture, for example, in university towns like Umeå [63]. Nevertheless, this might have affected the results. Third, we decided to dichotomize GDS-15 according to current guidelines for depression to make the results more clinically relevant. Also, PRISM-CC scores could not be treated as continuous due to the small proportion of individuals with low scores (more self-management difficulty). However, because of that, some information could have been lost.

## Conclusion

This study highlights the importance of a comprehensive, individualized approach to self-management support for older adults, particularly addressing both physical and emotional challenges. These findings underscore the need for regular mental health screenings and tailored self-management support in primary health care settings. The PRISM-CC can be used to identify older adults in need of self-management support and help older adults communicate their difficulties, enabling individualized self-management support. Future research should explore intervention strategies that integrate mental health support into self-management programs for individuals with long-term health conditions.

## Data Availability

The dataset generated and analysed during the current study is not publicly available because participant consent included restrictions on the use of the data due to patients’ privacy concerns. Limited availability is possible. Researchers wishing information may contact ÅA.

## Appendix 2. Domain definitions and items of the swedish version of the patient reported inventory of self-management of chronic conditions (PRISM-CC)

**Table:**

Resource	Self-perceived success in seeking, pursuing and/or managing needed formal or informal supports and resources.
Res1	When I have appointments with my healthcare providers, I tell them what I want or need.
	*När jag träffar mina vårdgivare berättar jag vad jag vill eller vad jag behöver.*
Res2	I talk to my healthcare provider(s) about my condition(s).
	*Jag talar med mina vårdgivare om min(a) sjukdomar. *
Res3	I arrange appointments with my health care provider(s).
	*Jag bokar in besök hos mina vårdgivare. *
Res4	When I need to, I find people to help me understand information I receive about my condition(s).
	*När jag behöver det hittar jag människor som kan hjälpa mig att förstå information jag får om min(a) sjukdomar. *
**Process **	**Self-perceived success in seeking information, being aware of choices and making good decisions. **
Pro1	I identify what information I can trust.
	*Jag tar reda på vilken slags information jag kan lita på. *
Pro2	I make informed decisions.
	*Jag fattar välgrundade beslut. *
Pro3	I think about the consequences of different decisions.
	*Jag tänker på konsekvenserna av olika beslut. *
Pro4	I try different things to find out what works best for me.
	*Jag provar olika saker för att ta reda på vad som fungerar bäst för mig.*
Pro5	I keep myself updated with new information related to my health conditions.
	*Jag håller mig uppdaterad med aktuell information om min(a) sjukdomar.*
**Internal **	**Self-perceived success in creating inner calm by preventing and managing stress, negative emotions, and internal distress. **
Int1	I set realistic expectations for myself.
	*Jag sätter realistiska förväntningar på mig själv.*
Int2	I accept the things I cannot change.
	*Jag accepterar saker jag inte kan förändra.*
Int3	I manage my emotions and reactions.
	*Jag hanterar mina känslor och reaktioner.*
Int4	I have and use ways to recover after a bad day.
	*Jag har och använder mig av olika sätt att återhämta mig efter en dålig dag.*
Int5	I deal with frustration caused by my health situation.
	*Jag hanterar frustration som orsakas av min hälsosituation.*
Int6	I manage my stress.
	*Jag hanterar min stress.*
Int7	I focus on the positives.
	*Jag fokuserar på det positiva. *
Int8	I forgive myself when I make a mistake.
	*Jag förlåter mig själv när jag gör misstag.*
**Activities **	**Self-perceived success in participating in everyday activities (leisure activities, work activities, household chores). **
Act1	I organize things in my home to make my life easier.
	*Jag organiserar saker i mitt hem för att underlätta mitt liv.*
Act2	I plan ahead before going somewhere to be sure I can manage my health condition(s).
	*Jag planerar i förväg innan jag ska åka någonstans för att vara säker på att jag kan hantera min(a) sjukdomar.*
Act3	I plan my time so I can get things done.
	*Jag planerar min tid så att jag kan få saker gjorda.*
Act4	I manage my health condition(s) so that I can do things I enjoy.
	*Jag tar hand om min(a) sjukdomar så att jag kan göra saker som ger mig glädje.*
Act5	I make time to do things I enjoy.
	*Jag avsätter tid för att göra saker som glädjer mig.*
**Social Interaction **	**Self-perceived success in disclosing health issues, managing social interactions and relationships. **
Soc1	I prioritize social interactions that I enjoy.
	*Jag prioriterar socialt umgänge som glädjer mig.*
Soc2	I can explain my symptoms so family and friends can understand them.
	*Jag kan förklara mina symptom så att familj och vänner kan förstå dem.*
Soc3	I clearly express my needs to others.
	*Jag uttrycker tydligt mina behov för andra. *
Soc4	I devote time and attention to those who are dear to me.
	*Jag lägger tid och uppmärksamhet på människor som jag bryr mig om.*
Soc5	When problems with my health arise, I stay in touch with people who are important to me.
	*När problem med min hälsa uppstår håller jag kontakten med människor som är viktiga för mig.*
**Healthy Behaviours**	**Self-perceived success maintaining a healthy lifestyle in order to enhance health and limit the risk of lifestyle related illness **
Hea1	I maintain healthy lifestyle behaviours that I know are important for my health.
	*Jag upprätthåller hälsosamma levnadsvanor som jag vet är viktiga för min hälsa.*
Hea2	I make healthy food choices.
	*Jag väljer att äta nyttigt.*
Hea3	I find ways to train my brain to keep mentally fit.
	*Jag hittar sätt att träna hjärnan för att hålla den igång.*
Hea4	I create healthy sleeping habits.
	*Jag skapar hälsosamma sömnvanor.*
Hea5S	I maintain healthy behaviours even when I have a lot to do.
	*Jag upprätthåller hälsosamma vanor även när jag har mycket att göra.*
**Disease Controlling **	**Self-perceived success in managing health conditions including managing medications and treatments, monitoring symptoms and limiting complications. **
Dis1	When problems with my health arise, I understand what to do to manage my condition(s).
	*När jag får problem med min hälsa förstår jag vad jag kan göra för att ta hand om min(a) sjukdomar. *
Dis2	I know what to do if I experience side-effects or other problems with my treatment or medication.
	*Jag vet vad jag ska göra om jag upplever biverkningar eller andra problem till följd av min behandling eller mina mediciner.*
Dis3	I know which symptoms I need to act upon.
	*Jag vet vilka symptom som jag behöver göra något åt.*
Dis4	I know what to do when my symptoms get worse.
	*Jag vet vad jag ska göra när mina symptom förvärras.*
